# Association of Menopausal Status, Expression of Progesterone Receptor and Ki67 to the Clinical Response to Neoadjuvant Chemotherapy in Luminal Breast Cancer

**DOI:** 10.1055/s-0039-3400457

**Published:** 2019-12

**Authors:** Leonardo Roberto da Silva, Renato Flora Vargas, Júlia Yoriko Shinzato, Sophie Françoise Mauricette Derchain, Susana Ramalho, Luiz Carlos Zeferino

**Affiliations:** 1 Universidade Estadual de Campinas, Campinas, SP, Brazil

**Keywords:** antineoplastic agents, breast neoplasms, segmental mastectomy, neoadjuvant therapy, estrogen receptors, agentes antineoplásicos, neoplasias da mama, mastectomia segmentar, terapia neoadjuvante, receptores estrogênicos

## Abstract

**Objective** To identify the biomarkers of response to neoadjuvant chemotherapy in early luminal breast cancer.

**Methods** A cross-sectional study that included all patients with early or locally-advanced luminal breast cancer submitted to neoadjuvant chemotherapy between 2013 and 2014. Demographic, clinic and pathologic data were retrieved from patient records. The expressions of the estrogen receptor (ER), the progesterone receptor (PR), and Ki67 were analyzed by immunohistochemistry (IHC). The status of the human epidermal growth factor receptor 2 (HER2) was evaluated by IHC and fluorescent in situ hybridization (FISH). Independent predictors of clinic and pathologic response were evaluated by stepwise logistic regression models and receiver operating characteristic (ROC) curve analysis.

**Results** Out of 298 patients identified, 115 were included in the analysis. Clinical complete response (cCR) was observed in 43.4% of the patients (49/113), and pathologic complete response (pCR) was observed in 7.1% (8/115) of the patients. The independent predictors of cCR were premenopausal status (*p* < 0.001), low PR expression (≤ 50% versus > 50%; *p* = 0.048), and Ki67 expression ≥ 14% (versus < 14%; *p* = 0.01). Patients with cCR were more commonly submitted to breast conserving surgery (34.7% versus 7.8%; *p* < 0.001). Increasing cut-off points for Ki67 expression were associated with an increase in specificity and a decrease in sensitivity to identify patients with cCR.

**Conclusion** Premenopausal status, lower PR expression and higher Ki67 expression were associated with a higher rate of cCR to neoadjuvant chemotherapy in luminal breast cancer.

## Resumo

**Objetivo** Identificar biomarcadores de resposta à quimioterapia neoadjuvante em câncer luminal de mama.

**Métodos** Estudo transversal em que foram incluídas todas as pacientes com câncer luminal de mama em estádio inicial ou localmente avançado que foram submetidas a quimioterapia neoadjuvante nos anos de 2013 e 2014. Dados demográficos, clínicos e patológicos foram obtidos de prontuários médicos. As expressões de receptor de estrogênio (RE), de receptor de progesterona (RP), e de Ki67 foram avaliadas por imuno-histoquímica (IHQ). A expressão do receptor tipo 2 do fator de crescimento epidérmico humano (*human epidermal growth factor receptor 2*, HER2) foi avaliada por IHQ e hibridização in situ por imunofluorescência (HISI). Análises de regressão logística e de curva de característica de operação do receptor (COR) foram usadas para investigar fatores preditivos independentes de resposta clínica e patológica.

**Resultados** De 298 pacientes identificadas, 115 foram incluídas na análise. Resposta clínica completa (RCc) foi observada em 43.4% das pacientes (49/113), e resposta patológica completa (RPc), em 7.1% (8/115). Os fatores preditivos independentes de RCc foram status menopausal (*p* < 0.001), baixa expressão de RP (≤ 50% versus > 50%; *p* = 0.048), e expressão de Ki67 ≥ 14% (versus < 14%; *p* = 0.01). Pacientes com RCc apresentaram maior probabilidade de serem submetidas a cirurgia conservadora da mama (34.7% versus 7.8%; *p* < 0.001). Aumento no ponto de corte para expressão de Ki67 foi associado a aumento da especificidade e redução da sensibilidade na identificação de pacientes com RCc.

**Conclusão** Status premenopausal, baixa expressão de RP e maior expressão de Ki67 estiveram associados a maior taxa de RCc à quimioterapia neoadjuvante no câncer luminal de mama.

## Introduction

Breast cancer is a heterogeneous group of diseases that differ in terms of behavior, prognosis and response to treatment.^1^ ^2^ Traditional prognostic and predictive markers, such as tumor size, lymph-node involvement, vascular invasion, and expression of estrogen receptor (ER), progesterone receptor (PR), and human epidermal growth factor receptor 2 (HER2) are used to select the treatment. However, these factors have limited power to define the prognosis and individualize treatment.^3^ ^4^ Neoadjuvant treatment for breast cancer has become an important strategy to downstage inoperable tumors, evaluate tumor biology, and identify potential biomarkers in a relatively short period of time.^5^ Pathologic complete response (pCR) after neoadjuvant chemotherapy (NAC) is considered a surrogate endpoint for long-term outcomes.^6^ ^7^ However, pCR is rarely seen in hormone-receptor-positive (luminal) breast cancer, and its prognostic impact is not clear.^8^ ^9^ Still, a subgroup of luminal tumors is chemo-sensitive.^10^ ^11^ There is a need to identify predictive factors that could help select patients with luminal breast cancer who would benefit from NAC.

The aim of the present study was to evaluate the association between patient characteristics, expression of ER, PR, HER2 and Ki67, and the clinicopathological response to NAC in patients with luminal breast cancer.

## Methods

**Fig. 1:**
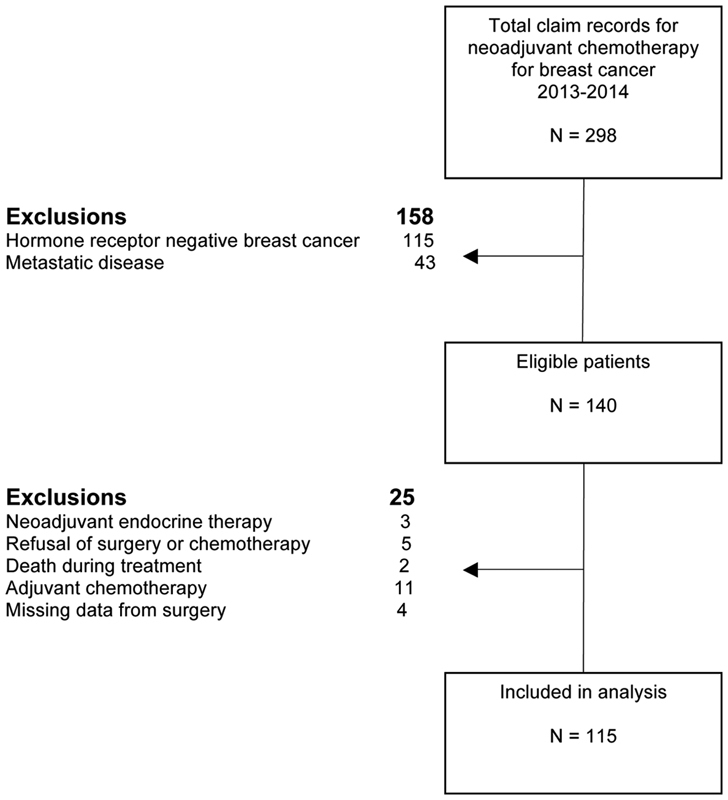
Flowchart of the patient selection process.

The present was a cross-sectional study conducted at Hospital da Mulher Professor José Aristodemo Pinotti, Centro de Atenção Integral à Saúde da Mulher (CAISM), Universidade Estadual de Campinas (UNICAMP), Brazil. The study was approved by the Research Ethics Committee of the School of Medical Sciences at UNICAMP (CEP 1246/2009). We reviewed the medical records of 298 patients submitted to NAC between January 2013 and December 2014, and 115 patients were included (Fig. 1). The inclusion criteria were diagnosis of invasive hormone-receptor-positive breast carcinoma, clinical stages I-III, and use of at least one cycle of NAC followed by surgery.

The tumors were histologically classified according to the World Health Organization criteria, the histological grade was determined according to the modified Bloom–Richardson system, and the tumors were grouped as low-to-moderate grade (grades I-II) and high grade (grade III).^12^ ^13^ We defined pCR as the absence of invasive disease on the breast and axilla.^14^ Immunohistochemistry was used to evaluate the expression of the ER (clone 1D5, 1:1 000, Dako, Carpinteria, CA, US), PR (clone PR 636, 1:800, Dako), HER2 (Clone PN2A, 1:1100, Dako), and Ki67 (clone MIB1, 1:500, Dako) protein using standard protocols. The ER and PR staining were classified as positive if at least 1% of the nuclei stained.^15^ The expression of Ki67 was reported as an average expression percentage from hot spots.^16^ Human epidermal growth factor receptor 2 staining was scored as 0 +/1+ (negative), 2+ (equivocal), or 3+ (positive). Equivocal cases were further confirmed by in situ hybridization, according to the recommendations of the American Society of Clinical Oncology/College of American Pathologists (ASCO/CAP).^17^

Tumor staging was defined according to the American Joint Committee on Cancer (AJCC) tumor-node-metastasis (TNM) cancer staging system (AJCC Cancer Staging Manual, 7th edition).^13^ The patients were grouped as IA-IIA (T_1-2_ N_0-1_), IIIB-IIIC (T_4_ N_0-3_), and N2-N3 for analytic purposes. Regarding the treatment protocol, 108 patients received anthracycline (AC) plus taxane, 4 patients received only AC for 6 cycles, and 2 patients, only taxane for 4 cycles. One patient received 5 cycles of CMF (C: cyclophosphamide 600 mg/m^2^; M: methotrexate 40 mg/m^2^; F: 5-fluorouracil 600 mg/m^2^) followed by 4 cycles of AC and 4 cycles of taxane. The clinical response was determined by caliper measurement of the largest tumor diameter at each visit, and it was classified as: partial (cPR) when there was incomplete reduction in dimension; complete (cCR) when there was no palpable lesion; stable disease when the dimensions were maintained; and progression when an increase in size occurred. For the statistical analyses, we considered clinical response (complete versus non-complete) as the response obtained in the primary tumor, since the degree of this response would directly impact on the decision regarding breast conservation.

The categorical variables were compared using the Chi-squared test or the Fisher exact test. Numerical variables with a non-gaussian distribution were analyzed by the Mann–Whitney U test. Receiver operating characteristic (ROC) curves were plotted to analyze the performance of potential predictors of clinical and pathological response, and the best cut-off points were determined according to Youden J statistics. A stepwise regression model was used to identify the independent predictors of response to treatment. The independent predictors are presented as the magnitude of the association (odds ratio, OR), and the respective 95% confidence intervals (95% CIs). Patients lost to follow-up were censored at the date of the last visit. Values of *p* ≤ 0.5 were required for significance in all of the analyses. The statistical tests were performed using the Statistical Analysis System (SAS, SAS Institute Inc., Cary, NC, US) software, version 9.4.

## Results

Pathological response data were retrieved for 115 patients (100%) and clinical response data were obtained for 113 patients (98.3%). Out of these 113 patients, 43.4% (49/113) showed cCR, and 7.1% (8/113) showed pCR. The median time from diagnosis to surgery was of 240.5 days. Most patients (91/115; 79,13%) underwent mastectomy, but cCR was associated with a higher rate of breast-conserving surgery (34.7% versus 7.8% for non-cCR; *p* < 0.001). Clinical complete response (cCR) occurred more frequently in younger patients (*p* = 0.01), premenopausal patients (*p* < 0.001), in cases of tumors with high histological grade (III versus I-II; *p* = 0.008), earlier clinical stage (*p* = 0.04), and higher expression of Ki67 (≥ 14% versus < 14%; *p* = 0.005). The mean percentage of cells showing ER expression was of 66% in tumors with cCR, and of 75% in those with non-complete response (*p* = 0.03); the corresponding values for PR expression were of 20% and 40% respectively (*p* = 0.025). Pathologic complete response was observed more frequently in cases of tumors with high histological grade (*p* < 0.001), earlier clinical stage (*p* = 0.002), and in HER2-positive tumors (*p* = 0.02) (Table 1). All tumors with pCR (*n* = 8) were high-grade ductal carcinomas with high Ki67 expression (≥ 14%) (Table 2).

**Table 1:** Baseline characteristics, demographic data and correlation with clinical and pathologic response

Baseline characteristic/demographic variable	Overall	Clinical complete response (cCR)		*p*-value	Pathologic complete response (pCR)		*p*-value
		Yes	No		Yes	No	
	115	49 (43.4)	64 (56.6)		8 (7.1)	107 (92.9)	
Age (median, years)	51.2	47.5	53.5	0.01^a^	43.0	50.0	0.08^a^
Menopausal status				< 0.001^b^			0.28^c^
Premenopausal	60 (52.2)	35 (71.4)	24 (37.5)		6 (75.0)	54 (50.5)	
Postmenopausal	55 (47.8)	14 (28.6)	40 (62.5)		2 (25.0)	53 (49.5)	
Histological subtype				0.53^c^			1.0^c^
Ductal	98 (86.7)	42 (85.7)	54 (87.1)		8 (100.0)	90 (85.7)	
Lobular	9 (8.0)	3 (6.1)	6 (9.7)		0 (0)	9 (8.6)	
Mixed	6 (5.3)	4 (8.2)	2 (3.2)		0 (0)	6 (5.7)	
Histological grade				0.008^b^			< 0.001^c^
I–II	83 (74.1)	31 (63.3)	52 (85.2)		0 (0)	83 (79.8)	
III	29 (25.9)	18 (36.7)	9 (14.8)		8 (100.0)	21 (20.2)	
Initial Tumor staging				0.06^b^			0.04^c^
T2	44 (39.3)	22 (45.9)	22 (34.4)		3 (37.5)	41 (39.4)	
T3	31 (27.7)	16 (33.3)	15 (23.4)		5 (62.5)	26 (25.0)	
T4	37 (33.0)	10 (20.8)	27 (42.2)		0 (0)	37 (35.6)	
Initial Nodal staging				0.43^b^			1.00^c^
N0	30 (26.1)	16 (32.6)	14 (21.9)		2 (25.0)	28 (26.2)	
N1	61 (53.0)	24 (49.0)	36 (56.2)		4 (50.0)	57 (53.3)	
N2–3	24 (20.9)	9 (18.4)	14 (21.9)		2 (25.0)	22 (20.5)	
ER status				0.19^c^			1.0^c^
Negative	3 (2.6)	2 (4.1)	0 (0)		0 (0)	3 (2.8)	
Positive (≥ 1%)	112 (97.4)	47 (95.9)	64 (100.0)		8 (100.0)	104 (97.2)	
Cells with ER expression: mean, % (SD),	71.5 (14.1)	66.0 (27.2)	75.0 (22.3)	0.03^a^	63.1 (29.6)	71.7 (24.4)	0.30^a^
PR status							0.62^c^
Negative	19 (17.0)	11 (23.4)	8 (12.5)	0.13^b^	2 (25.0)	17 (16.4)	
Positive (≥ 1%)	93 (83.0)	36 (76.6)	56 (87.5)		6 (75.0)	87 (83.6)	
Cells with PR expression: mean, % (SD),	41.5 (14.1)	32.4 (31.8)	46.3 (31.7)	0.025^a^	29.4 (27.3)	41.4 (32.7)	0.34^a^
HER2 status				0.05^b^			0.02^c^
Negative	87 (76.3)	32 (66.7)	53 (82.8)		3 (37.5)	84 (79.3)	
Positive	27 (23.7)	16 (33.3)	11 (17.2)		5 (62.5)	22 (20.7)	
Pretreatment Ki67 index				0.005^b^			0.10^c^
< 14%	32 (29.6)	6 (14.0)	25 (39.1)		0 (0)	32 (32.0)	
≥ 14%	76 (70.4)	37 (86.0)	39 (60.9)		8 (100.0)	68 (68.0)	

Abbreviations: ER, estrogen receptor; HER2, human epidermal growth factor receptor 2; PR, progesterone receptor; SD, standard deviation.

Notes: Data are presented as numbers and percentages, unless otherwise indicated.

a Mann-Whitney test.

b Chi-squared test.

c Fisher exact test.

* Missing data: histological subtype (*n* = 2; 0.02%); histological grade (*n* = 3; 0.03%); initial tumor (T) staging (*n* = 3; 0.03%); PR (*n* = 3; 0.03%); HER2 status (*n* = 1; 0.008%); pretreatment Ki67 (*n* = 7; 0.06%); clinical complete response (*n* = 2; 0.02%); percentage of cells with ER expression (*n* = 2; 0.02%); percentage of cells with PR expression (*n* = 2; 0.02%).

**Table 2:** Baseline characteristics and demographic data of patients with pCR

Patient	Age	Menopausal status	Tumor staging	Nodal staging	Histological subtype	Histological grade	HER2	Pretreatment Ki67	Treatment
1	48	pre	T3	N1	ductal	III	negative	80	AC-T
2	50	pre	T2	N2	ductal	III	positive	70	AC-T
3	56	post	T2	N0	ductal	III	negative	70	AC-T
4	49	post	T3	N2	ductal	III	positive	30	AC-T
5	40	pre	T3	N1	ductal	III	positive	20	AC-T
6	43	pre	T3	N1	ductal	III	positive	15	AC-T
7	40	pre	T3	N1	ductal	III	positive	20	AC-T
8	32	pre	T2	N0	ductal	III	negative	15	AC-T

Abbreviations: AC, doxorubicin plus cyclophosphamide; ER, estrogen receptor; HER2, human epidermal growth factor receptor 2; pCR, pathologic complete response; PR, progesterone receptor; T, paclitaxel or docetaxel.

**Fig. 2:**
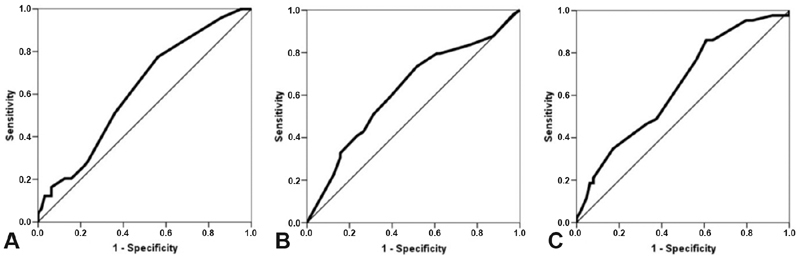
Receiver operating characteristic (ROC) curve analysis of estrogen receptor (ER), progesterone receptor (PR), and Ki67 expression as predictors of clinical complete response (cCR). (**A**) ER expression. The calculated area under the curve (AUC) was of 0.619 (95% confidence interval [95%CI]: 0.516–0.722; *p* = 0.03); a cut-off point of 85% had a sensitivity of 77.5% and a specificity of 43.8% to identify patients who are likely to develop cCR. (**B**) PR expression. The AUC was of 0.623 (95%CI: 0.518–0.728; *p* = 0.026); a cut-off point of 50% had a sensitivity of 73.5% and a specificity of 48.4%. (**C**) Ki67 expression. The AUC was of 0.642 (95%CI: 0.536–0.747; *p* = 0.013); a cut-off point of 14% had a sensitivity of 86.1% and a specificity of 39.1%.

The analysis of the ROC curve for ER expression as a predictor of cCR showed an area under the curve (AUC) of 0.619 (*p* = 0.03), and a cut-off point of 85% showed a sensitivity of 77.5%, a specificity of 43.8%, and an accuracy of 58.4%. For PR expression, the AUC was of 0.623 (*p* = 0.026), and a cut-off point of 50% showed a sensitivity of 73.5%, a specificity of 48.4%, and an accuracy of 59.3%. The Ki67 AUC was of 0.642 (*p* = 0.013), and a cut-off point of 14% showed a sensitivity of 86.1%, a specificity of 39.1%, and an accuracy of 57.9% (Fig. 2).

We tested the performance of different cut-off points for Ki67 expression in the identification of the cCR. A cut-off point of 10% was associated with a sensitivity of 95.35% and a specificity of 20.31%, while a cut-off point of 30% showed a sensitivity of 46.51% and a specificity of 67.19%. This analysis shows that increasing the cut-off point for Ki67 expression is associated with a gain in specificity and a reduction in sensitivity in detecting cCR (Table 3).

**Table 3:** Performance of different cut-off points of Ki67 expression to determine the cCR according to the ROC curve analysis

Ki67 cutoff	Sensitivity	Specificity	PPV	NPV
≥ 10% versus < 10%	95.35	20.31	44.57	86.67
≥ 14% versus < 14%	86.10	39.10	48.68	80.65
≥ 20% versus < 20%	76.74	43.75	47.83	73.68
≥ 30% versus < 30%	46.51	67.19	48.78	65.15

Abbreviations: CCR, clinical complete response; NPV, negative predictive value; PPV, positive predictive value; ROC, receiver operating characteristic.

Note: Data are presented as percentages.

Explanatory variables associated with the clinical and pathological response in the univariate analysis were subsequently tested on a multivariate stepwise regression model. For ER, PR and Ki67 expression, optimal cut-off points determined at the ROC curve analysis were used. The multivariate regression model did not identify independent predictors of pCR, including histological grade, clinical staging, and HER2 expression. Premenopausal patients (OR: 4.71; 95%CI: 1.9–11.7; *p* < 0.001), low expression of PR (≤ 50% versus > 50%; OR: 2.58; 95%CI: 1.01–6.62; *p* = 0.048), and higher expression of Ki67 (≥ 14% versus < 14%; OR: 3.92; 95%CI: 1.33–11.53; *p* = 0.01) were identified as independent predictors of cCR (Table 4).

**Table 4:** Stepwise regression model results for independent predictors of cCR

Explanatory variable	Odds ratio (95% CI)	*p*-value
Menopausal status: pre versus post	4.71 (1.9–11.7)	< 0.001
Mean PR expression: ≤ 50% versus > 50%	2.58 (1.01–6.62)	0.048
Ki67 expression: ≥ 14% versus < 14%	3.92 (1.33–11.53)	0.01

Abbreviations: 95%CI, 95% confidence interval; CCR, clinical complete response; PR, progesterone receptor.

In a subgroup analysis including only luminal HER2-negative tumors (*n* = 88), 33 (37.5%) patients presented cCR, which was associated with premenopausal status (*p* < 0.001) and higher pre-treatment Ki67 expression (≥ 14% versus < 14%; *p* = 0.0123). On the multivariate stepwise regression model, premenopausal status (OR: 1.90; 95%CI: 0.81–2.98; *p* < 0.001) and higher pre-treatment Ki67 expression (≥ 14% versus < 14%; OR: 1.60; 95%CI: 0.32–2.88; *p* = 0.014) were independent predictors of cCR (data not shown). Regarding the luminal HER2-negative tumors, there were 3 (3.4%) cases with pCR that were characterized by high histological grade (III versus I-II; *p* = 0.008) (data not shown).

## Discussion

In our study, we investigated the association between clinical and pathological parameters to predict cCR and pCR to NAC in luminal breast cancer. In the univariate analysis, younger age, premenopausal status, high histological grade and higher expression of Ki67 were associated with cCR. Pathologic complete response was observed in less than 10% of the patients, and all of their tumors were of ductal histology, with high histological grade and higher Ki67 expression. Premenopausal status and higher expression of Ki67 were independent predictors of cCR in luminal breast cancer irrespective of HER2 expression. Low expression of PR was an independent predictor of cCR only in luminal HER2-positive tumors. Our ROC curve analysis for ER, PR and Ki67 expression showed a moderate performance to identify tumors with cCR. Increasing cut-off points for Ki67 expression were associated with an increase in specificity and decrease in sensitivity to identify the cCR.

In our cohort of patients, objective clinical response was observed in 88.6% of tumors, with a cCR rate of 43.4% on the breast. These response rates are somewhat higher than what has been previously described.^18^ However, these differences may be explained by two main factors: firstly, in the present study, we classified the clinical response based exclusively on the clinical examination. Moreover, evidence shows that there is a poor correlation between the response evaluated by physical examination and imaging methods, and this may reflect the dynamics of tumor response to treatment.^19^ ^20^ Secondly, in the present study, 94% of the patients were treated with an anthracycline and a taxane, opposed to 74.9% in the American College of Surgeons Oncology Group (ACOSOG) Z1071 trial. Indeed, improvement in response rates obtained by the addition of a taxane to an anthracycline in the neoadjuvant setting is well documented.^21^ ^22^

Our results showed that premenopausal patients were more likely to achieve cCR. In several studies, young age at diagnosis was identified as an independent predictor of recurrence and mortality in breast cancer patients.^23^ ^24^ The poorer prognosis of these patients may be related to their higher likelihood of developing more aggressive tumors. In the Prospective Study of Outcomes in Sporadic Versus Hereditary Breast Cancer (POSH), 2.956 young patients (aged 40 years or younger) with a breast cancer diagnosis were recruited, and the study showed that 50.2% of the patients had a node-positive disease, 58.9% had high grade tumors, and 33.7% were ER-negative, factors associated with a higher response to chemotherapy.^25^ Similar findings were reported by other authors, along with high rates of lymphovascular invasion and lymphocytic infiltration.^23^ ^26^ Tumors within the same molecular subtype are heterogeneous, and, although the reasons for these differences are not clear, recent evidence suggests that tumors in younger patients show higher expression of genes related to mammary stem cells and deregulation of mitogen-activated protein kinase (MAPK) and phosphoinositide 3-kinase (PI3K) pathways, which can contribute to endocrine therapy resistance and chemosensitivity in ER-positive tumors.^27^ ^28^ ^29^ Tumors with lower PR expression had a higher probability of achieving cCR. In fact, it has been shown that PR expression in ER-positive tumors is associated with less aggressive phenotypes, and that tumors with lower PR expression may be less dependent on ER pathway signaling and show upregulation of the PI3K pathway.^30^ ^31^ ^32^ ^33^ ^34^

Ki67 expression was also an independent predictor of cCR, and higher expression levels are associated with a higher likelihood of acieving cCR. Xu et al^35^ evaluated 129 breast cancer patients submitted to NAC, and showed that tumors with Ki67 expression > 10% had better clinical response. The use of Ki67 as a predictive and prognostic marker is a matter of debate due to poor reproducibility.^16^ To date, different Ki67 expression cut-off points have been suggested to identify tumors with a higher probability of response to chemotherapy. As we have shown, the accuracy of different cut-off points is quite variable. Finding a unique cut-off point is unlikely, and this evaluation should consider the clinical scenario.^36^ Our ROC curve analysis showed that the expressions of Ki67, ER and PR have a modest ability to identify patients with cCR. But, despite the relatively poor predictive performance, the curves are plotted above the line of no discrimination, which implies a better classification than random results.

In the present study, the pCR rate was comparable with data published by other authors,^7^ ^11^ ^37^ reflecting the relative chemoresistance of ER-positive tumors. However, the pCR does not appear to have a prognostic impact on luminal breast cancer patients, especially among low-grade and HER2-negative tumors.^6^ ^37^ Although we could not identify independent predictors of pCR, all tumors with pCR were ductal, had high histological grade, Ki67 expression > 14%, and most were HER2-positive. Histological grade and Ki67 reflect tumor proliferation, and tumors with high proliferative activity are more sensitive to chemotherapy.^38^ Rates of pCR are particularly high among tumors that are HER2-positive, even in the absence of anti-HER agents.^6^ ^7^ ^37^ ^39^

The limitations of the present study include its retrospective nature, which exerted an impact on our ability to retrieve some data. We studied a small sample of patients, but other authors^11^ ^35^ reported on similar sample sizes. The small number of patients with pCR may have limited the identification of independent predictors for this outcome. One strength of our study is that the majority of our patients underwent a modern and standardized chemotherapy regimen using the most advanced cytotoxic agents available in neoadjuvant settings, especially in the context of limited access to anti-HER2 agents.

## Conclusion

In conclusion, patients with hormone-receptor-positive tumors show high rates of clinical response to NAC, and achieving cCR is associated with a higher probability of breast-conserving surgery. Premenopausal status, lower expression of PR and higher expression of Ki67 were associated with a higher likelihood of achieving cCR, and may be used to select patients with hormone-receptor-positive tumors who might benefit more from the NAC. This strategy has the potential of effectively reducing overtreatment and costs. Additional studies are necessary to better understand the underlying mechanisms of these associations.
